# Identification, pathogenicity and molecular characterization of a novel fowl adenovirus 8b strain

**DOI:** 10.1016/j.psj.2024.103725

**Published:** 2024-04-03

**Authors:** Yapeng Song, Lin Liu, Wenjie Sun, Wenming Gao, Xiaonan Song, Yang Wang, Qiang Wei, Zongmei Huang, Xinsheng Li

**Affiliations:** ⁎ College of Veterinary Medicine, Henan Agricultural University, Zhengzhou, 450002, China; † Henan Provincial Key Laboratory of Animal Immunology, Henan Academy of Agricultural Sciences, Zhengzhou, 450002, China

**Keywords:** fowl adenovirus, isolation, recombination, identification, pathogenicity

## Abstract

Since 2012, there has been a noticeable upward trend in the global incidence of inclusion body hepatitis (**IBH**) cases, leading to substantial economic losses in the poultry industry. In response to this trend, the current study aimed to investigate the phylogenetic information, genetic mutations, and pathogenicity of the highly pathogenic fowl adenovirus (**FAdV**) strain HN1472, which was isolated from liver samples obtained from a laying flock affected by IBH. This investigation was carried out using 1-day-old specific pathogen-free (**SPF**) chickens. Recombination and phylogenetic analyses confirmed that HN1472 is a recombinant strain derived from FAdV-8a and FAdV-8b, and exhibited significant genetic divergence in the hexon, fiber, and ORF19 genes. Notably, the phylogenetic analysis identified recombination events in these regions. Furthermore, animal experiments revealed that HN1472 is a highly pathogenic isolate, causing 80% mortality and manifesting clinical signs of IBH in SPF chickens. Furthermore, the recombinant FAdV serotype 8b (**FAdV-8b**) was found to be widely distributed in various tissues, with a higher concentration in the livers and gizzard tissue at 3 d postchallenge (**dpc**). Collectively, these findings contribute to our current understanding of the factors influencing the pathogenicity and genetic diversity of FAdV serotype 8b (**FAdV-8b**) in China.

## INTRODUCTION

Fowl adenoviruses (**FAdV**) are nonenveloped icosahedral viruses composed of linear double-stranded DNA with size ranging from 25 to 46 kb (Harrach et al., 2019). They belong to the family *Adenoviridae*, genus *Aviadenovirus*, and are further classified into 5 species (FAdV-A to E) with 12 serotypes (FAdV-1 to 8a, 8b to 11), according to the guidelines of the International Committee on Taxonomy of Viruses (Hess and M., 2000; Harrach et al., 2011; Shah et al., 2017). FAdVs have a global distribution and are considered as an economically important disease in various countries. They are capable of infecting all domestic avian species across different age groups.

FAdVs are avian viruses primarily infecting young broiler chickens and can be isolated from apparently healthy birds (Adel et al., 2021). Poultry infected with FAdVs may exhibit various clinical signs, predominantly causing 3 disease complexes in chickens: naturally acquired outbreaks of inclusion body hepatitis (**IBH**), caused by FAdV-D (serotypes 2 and 11) and E (serotypes 8a and 8b), hepatitis-hydropericardium syndrome (HHS), caused by FAdV-C (serotype 4), and gizzard erosions (**GE**), caused by FAdV-A (serotype 1) (Hess, 2016; Schachner et al., 2018). Although all 12 FAdV serotypes have been associated with IBH outbreaks, the most prevalent strains belong to serotypes within the species of FAdV-D and FAdV-E, which have been isolated in multiple countries (Schachner et al, 2016). When comparing the genetical characteristics of different FAdV virus strains isolated from AGE, a closer molecular relationship is observed between the pathogenic types of HHS and IBH, thereby indicating a shared pathogenic and immune mechanism (Schachner et al., 2018; De Luca et al., 2022).

The outbreaks of IBH have had significant economic implication for the global poultry industry. The characteristic features of IBH include the presence of necrotic foci in a pale hemorrhagic liver, along with basophilic intranuclear inclusion bodies (Dar et al., 2012). While generally not severe, IBH can lead to decreased in performance and an increased mortality rate ranging from 10 to 30% due to secondary infections (Mittal et al., 2014; Changjing et al., 2016). Reports have indicated that IBH outbreaks can result from both vertical and horizontal transmission. The precise pathogenesis of FAdV-induced diseases remains elusive, as different strains pathogenicity may vary within the same serotype. Furthermore, the immune system status of avian, along with concurrent infections by other immunosuppressive agents, can significantly impact the occurrence and progression of viral infection (Kabanova et al., 2022; Abdel-Alim et al., 2023).

Phylogenetic analysis revealed the global distribution of FAdV-8 without any geographical correlation (Niczyporuk and Samanta, 2016). Meanwhile, there is a relative lack of molecular characterization for other potentially devastating FAdVs that have been circulating in China for a long time. In this study, we isolated and characterized a FAdV strain named HN1472 from the livers of deceased chickens, and evaluated pathogenicity in specific pathogen-free (SPF) chickens. Our findings offer valuable insights into the molecular epidemiological surveillance of genetically recombined FAdV and the development of effective control strategies.

## Materials and Methods

### Sample Collection, Cells, and Animals

Liver tissue samples were collected from 140-d-old Hy-Line Brown commercial layer chickens in Zhoukou, Henan Province, China in 2021. The egg production rate of infected chickens increased slowly, while the mortality rate reached 10% within 3 weeks. FAdV vaccines had not been vaccinated before. The postmortem examination revealed typical hepatic changes indicative of IBH in the affected and deceased chickens. Chicken Leghorn male hepatocellular (**LMH**, ATCC CRL-2117) cells were cultured in DME/F-12 (HyClone) supplemented with 10% fetal bovine serum, 100 IU/mL penicillin, and 100 µg/mL streptomycin. The cells were incubated at 37°C in a humidified 5% CO_2_ incubator. SPF white leghorn chickens were purchased from Boehringer Ingelheim Biotechnology Co. Ltd (Beijing, China). These chickens were housed in SPF isolators and maintained under negative pressure.

### Virus Isolation

Liver tissue samples were homogenized in phosphate-buffered saline (**PBS**), followed by filtration through a Millipore syringe filter with a pore size of 0.22 μm to produce an inoculum for viral isolation after undergoing 3 freeze-thaw cycles. Afterwards, the resulting supernatant was used to infect LMH cells grown as monolayer in DME/F-12 medium (Gibco) supplemented with 10% fetal bovine serum at 37°C under humidified conditions containing 5% CO_2_. The cells were observed daily to monitor any cytopathic effects. When 80% cytopathies were observed, both the supernatant and infected cells underwent 3 additional freeze-thaw cycles prior to collection. After 3 round of plaque purification procedure and exogenous pathogenic microorganism detection, the FAdV-positive supernatants were subsequently amplified on confluent monolayers of LMH cell. The purified strains were identified using PCR to rule out other viruses such as AIV, IBV, IBDV, NDV, FAdV-4, and CIAV.

### Verification of Viral DNA

The identification of FAdV involved conducting a PCR assay to detect the FAdVs Hexon gene using specific primers (Silaen et al., 2020). Verification of the presence of FAdVs in the supernatant was achieved through PCR targeting an 897nt fragment of the hexon gene, utilizing the primers FAdV-F: 5′-CAAGTTCAAGGGAGACGGTGGT -3′; and FAdV-R: 5′-TAGTGATGCCTGGACATCAT-3′. The PCR reactions were carried out as follows: initial denaturation at 95°C for 4 min, followed by 35 cycles at 95°C for 15 s, annealing at 56°C for 30 s, extension at 72°C for 30 s, and a final elongation step at 72°C for 10 min. Primer synthesis and PCR product sequencing were performed by Sangon Biotech (Shanghai, China).

### Determination of 50% Tissue Culture Infection Doses

The LMH cells were initially seeded into each well of a 96-well cell culture plate, with 10^5^ cells in each well. Following an overnight incubation period, virus stock solutions ranging from 10^−1^ to 10^−9^ were used to infect the cells, with each dilution being repeated in 8 wells. Additionally, an equal number of wells were designated for the negative control. Following an hour-long incubation of the infected cells at 37°C in a 5% CO_2_ environment, the culture supernatant was removed and the cells were washed twice with PBS. Subsequently, 0.1 mL of DME/F12 medium supplemented with 2% FBS was added, and the cells were then incubated for 7 days. Finally, the Reed-Muench method was employed to calculate the 50% tissue culture infective dose (**TCID_50_**) after the incubation period.

### Transmission Electron Microscopy

The virus particles were observed using transmission electron microscopy (**TEM**) according to previously described methods (Wang et al., 2019). The purified virus pellets were resuspended in PBS buffer and subjected to negatively staining with 1% phosphotungstic acid for 1 min. After blotting and drying, the grids were examined using a TEM (JEM-1400, JEOL Ltd., Japan).

### Complete Genome Sequencing

The DNA from the disease venom was extracted using a DNA extraction kit in accordance with the given protocol. After consulting the whole genome sequence of FAdV-8b 764 (GenBank: KT862811) available in GenBank, 19 primer pairs were designed for the amplification of the entire genome of the HN1472 isolate (see Table 1). To differentiate between recombinant and mixed strains, primers 8a-F/R were designed to amplify the fiber gene (see Table 1). Each 25 μL PCR reaction volume was constituted of 12.5 μL 2 × Rapid Taq Master Mix (Vazme Biotech, China), 2 μL total DNA from HN1472, 0.5 μL of each primer (10μmol/L), and nuclease-free water to a total volume of 25 μL. Subsequently, the PCR products were sequenced by Sangon Biotech (Shanghai, China) and manually assembled using the DNAMAN program.

**Table 1:** The primers for whole genome sequencing of HN1472.

Primer Name	Sequences
E1	F: 5′-CATCATATATATATACCTGCTTAAAAT-3′ R: 5′-CCCTTCCGCCTTATTATCACT-3′
E2	F: 5′-GACTTTGACTTTTTCAAGTGAAT-3′ R: 5′-CCCCAAATTCCATCTCAT-3′
E3	F: 5′-ACGGAGCCTTGTGTCGTGTGGG-3′ R: 5′-AGAGTTCTACAAGCGGGTCACGGGG-3′
E4	F: 5′-TCCAAGCGGTAGAGCAGAG-3′ R: 5′-TGAACGCCGAGCCTAAAA-3′
E5	F: 5′-CGATGTTCATGTCGTCGTGGAG-3′ R: 5′-AAAACCCACTTCAAATCCTTCAAC-3′
E6	F: 5′-TGCTGCCGACGAGGAAGTTG-3′ R: 5′-ACTCTTTTGGACCTGCCTCACCGAT-3′
E7	F: 5′-GTCCCCTGAGATGAACGT-3′ R: 5′-CGCAGCACGTTGTTTTG-3′
E8	F: 5′-ATTCAACTGGACAACCGAT-3′ R: 5′-GATATTTGCCGAACTGATG-3′
E9	F: 5′-CGGTTTGCCAAGGTGTCG-3′ R: 5′-CGCCGAAGAGGCTGAGAT-3′
E10	F: 5′-TGCGGATACGGGAAAC-3′ R: 5′-CGGTCCCATTGTTCTCG-3′
E11	F: 5′-CCACTTGATTGTGAAAGGAG-3′ R: 5′-TTCCGAGGCTGTAAATGG-3′
E12	F: 5′-GAGCAACTACGCCACCTACC-3′ R: 5′-AGAGCACCGTCAGCAACG-3′
E13	F: 5′-CAGGGACCCAGACAAGT-3′ R: 5′-GAGGGATCGCTGGACATC-3′
E14	F: 5′-GATGTCCAGCGATCCCTC-3′ R: 5′-TGGAGCAGGCTGGAAGG-3′
E15	F: 5′-CGGTGTTGTGCGGAGGTA-3′ R: 5′-CCTTGTTCGGTTTCCCTC-3′
E16	F: 5′-TAATGAGGGTTTCAAAGTGGG-3′ R: 5′-GCAACAGGCAAACATCATAAACACG-3′
E17	F: 5′-CTGCTTGAGTACGGGTGAGTATGT-3′ R: 5′-TATGCGGTCTCCCAATTTCAACG-3′
E18	F: 5′-CCATAGGCTACAATGGAATACTGCC-3′ R: 5′-CGGATAGAAGAATAAGGGCTAAGATGG-3′
E19	F: 5′-CATCTTAGCCCTTATTCTTCTATCCG-3′ R: 5′-CATCATATATATATACCTGCTTAAA-3′
8a-F	F: 5′-GAGCCTGCGACTCCATCTCCGAC-3′
8a-R	R: 5′-CAGTAGCCCTTTACGTTGTGTTG-3′

### Phylogenetic Analysis

To assess nucleotide and amino acid sequence identity, as well as type/species classification, a phylogenetic analysis was performed using MEGA 7.0 software. The analysis involved constructing a phylogenetic tree based on complete genes (Hexon and fiber gene) from the newly plaque-purified isolation strains and prototype sequences representing various genera for each existing serotype, using the neighbor-joining method. In addition, adenovirus species A-E sequences were extracted from the GenBank database for inclusion in this study. The resulting phylogenetic tree was constructed using the neighbor-joining method and bootstrap values were determined through 1,000 original data replicates. Following this, recombination analysis between the new isolate and its 5 parental strains was conducted using SimPlot version.

### Pathogenicity Analysis of HN1472 Isolate in SPF Chickens

In order to assess the pathogenicity of HN1472, 20 one-day-old SPF chickens were evenly divided into group A and B. Group A was intramuscularly inoculated with a dose of 10^5.8^ TCID_50_ of strain HN1472, while group B served as uninfected controls and received the same amount of PBS. Each group of chicks was housed in individual negative-pressure isolators with controlled environments with free intake to water and food.

Daily observations for 14 d postchallenge (**dpc**) focused on documenting food intake, mental state, and death, while also monitoring weight fluctuations. Virus shedding assessment was conducted by collecting oral-pharyngeal and cloacal samples at 1, 3, 5, 7, 9, 11, and 14 dpc, which were then immersed in a solution of 1 mL PBS buffer for detection purposes. Additionally, tissue samples (lung, bone marrow, thymus, kidney, liver, duodenum, caecum, rectum) were collected from group A chickens after their demise to assess the viral load. Concurrently, tissue samples (heart, liver, kidney, spleen) were promptly immersed in a 10% neutral-buffered formalin solution for fixation. Five chickens from group B were euthanized, and the corresponding tissues were collected and preserved at 5 dpc. Subsequently, following a 24-h fixation period, the tissue samples underwent processing, including embedding in paraffin wax, staining with hematoxylin and eosin (HE), before observation using standard light microscopy.

### Real-Time PCR

The real-time PCR method was utilized for virus quantification, measuring the DNA copy number of FAdV in tissues and cloacal swabs by using the standard curve. The primers used were Hex-F 5′-TAGACACCACCGCACAGAAATAC-3′ as the forward primer, Hex-R 5′-TGCCTGACCGTTCGGAGTT-3′ as the reverse primer, and P: 5′- CCAACTTACATCGGGTTCCGTGACAAT-3′ as Taqman probe. The qPCR test was carried out on LineGene 9600Plus (BIOER, China) with the reaction conditions including an initial denaturation at 95°C for 2 min, followed by 30 cycles at 95°C for 30 s, 55°C for 1 min, and 72°C for 6 min. Subsequently, a final extension step was performed at 72°C for 10 min. The amplified Hexon gene fragment was cloned into the pMD-19T vector (TaKaRa, Japan) for further analysis. Samples were evaluated based on the standard curve, with a fluorescence signal surpassing a predefined threshold limit at the cycle threshold (Ct) considered positive if detected before 36 cycles. This criterion ensured primer specificity and excluded cross-reactivity with other pathogens.

### Histopathology

The liver and spleen samples were collected and immersed in a 10% formaldehyde solution for subsequent histopathological examination. After fixation in 10% neutral-buffered formalin, the samples were then routinely processed, including embedding in paraffin block and sectioning on a microtome at 4 μm. Subsequently, the cut sections were stained using haematoxylin-eosin method (H&E) and examined using light microscopy to identify any histopathological lesions. The histopathological changes were scored at 5 dpc. A score of 0 was assigned to samples with no noticeable lesions, while scores of 1, 2, and 3 were given to samples with slight, moderate, and severe lesions, respectively.

### Statistical Analysis

The statistical analysis of data was conducted using GraphPad Prism 8.3.0 software. The results were expressed as mean ± standard deviation (Mean ± SD), and the differences among the test groups were compared by one-way analysis of variance (**ANOVA**) (Oksanich et al., 2007). A *p*-value of < 0.05 was considered to indicate statistically significant.

## RESULTS

### Identification and Characterization of FAdV-8b Variant Isolate

**Figure 1:**
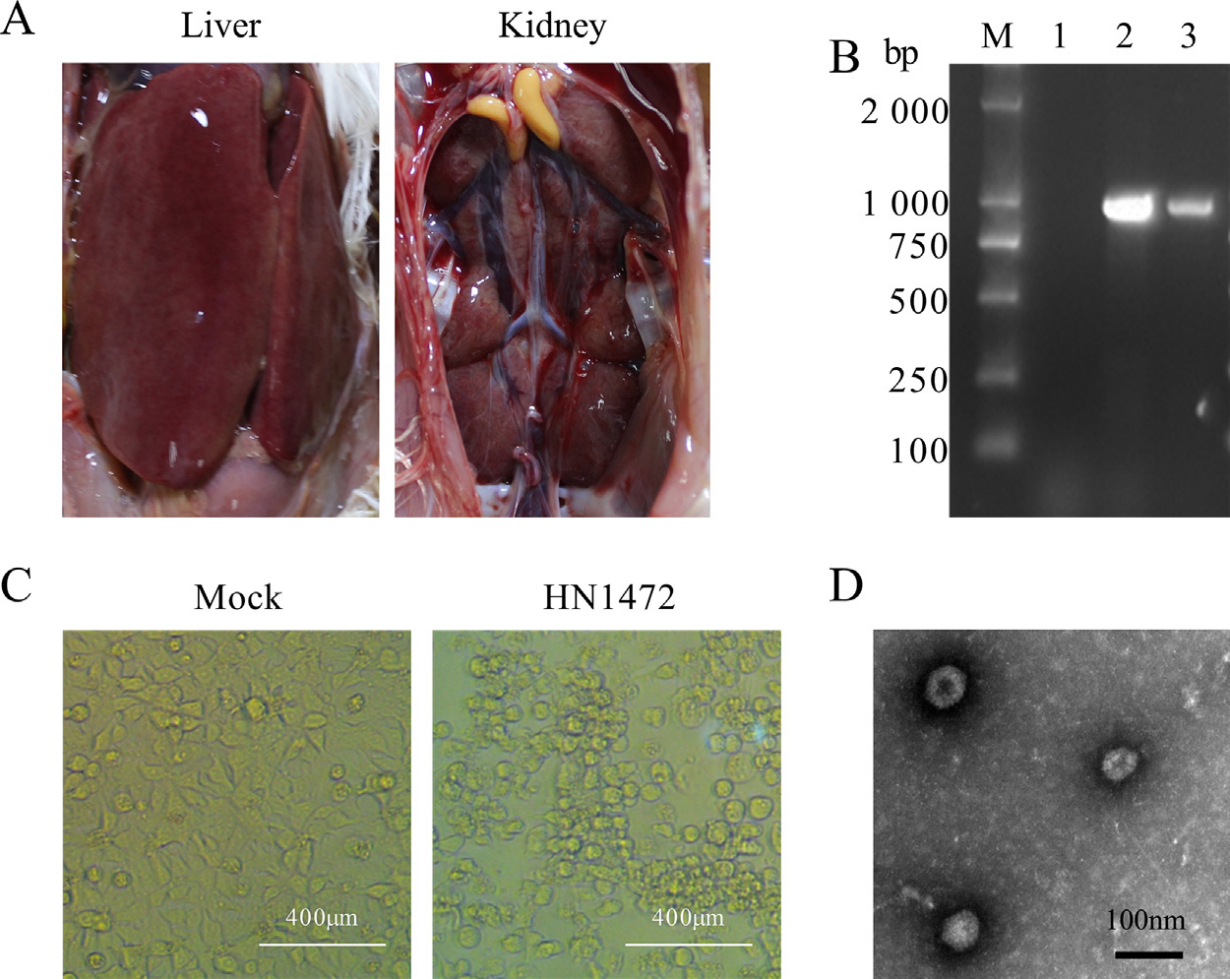
Characterization of variant isolates of the FAdV HN1472 strain. (A) Prominent pathological changes observed in chickens infected with FAdV HN1472-infected chickens. (B) Amplification results of the partial hexon gene. (C) Cytopathic effect caused by the FAdV HN1472 strain. (D) Transmission electron microscopy visualization of HN1472 viral particles.

In June 2021, a significant mortality of chickens was observed at the laying chicken farm in Zhoukou, Henan Province. Necropsy examinations of the deceased chickens revealed typical symptoms of IBH, including hepatomegaly, hemorrhage, and kidney enlargement (Figure 1A). Liver samples from the infected chickens were collected and homogenized with PBS to detect the presence of FAdVs. The resulting homogenate was then inoculated into LMH cells. The initial introduction of this inoculated mixture into cell cultures visibly impacted the LMH cells. At 48 h postinfection, compared to the control, the LMH cells exhibited became rounder morphology, increased refractivity, aggregation into grape-like clusters, and partial disintegration (Figure 1C). After 3 consecutive passages, an 897 nt region of the FAdV hexon gene was amplified using the PCR technique and subsequently subjected to sequencing (Figure 1B). Following plaque purification and multiple passages, FAdV HN1472 strain was successfully isolated. The virus titer of HN1472 was determined to be 10^5.8^ TCID_50_/0.1mL. The virus particles of HN1472 as classical adenovirus were observed using TEM (Figure 1D).

### Analysis of Complete Sequences of FAdV-8b HN1472 Isolates

**Figure 2:**
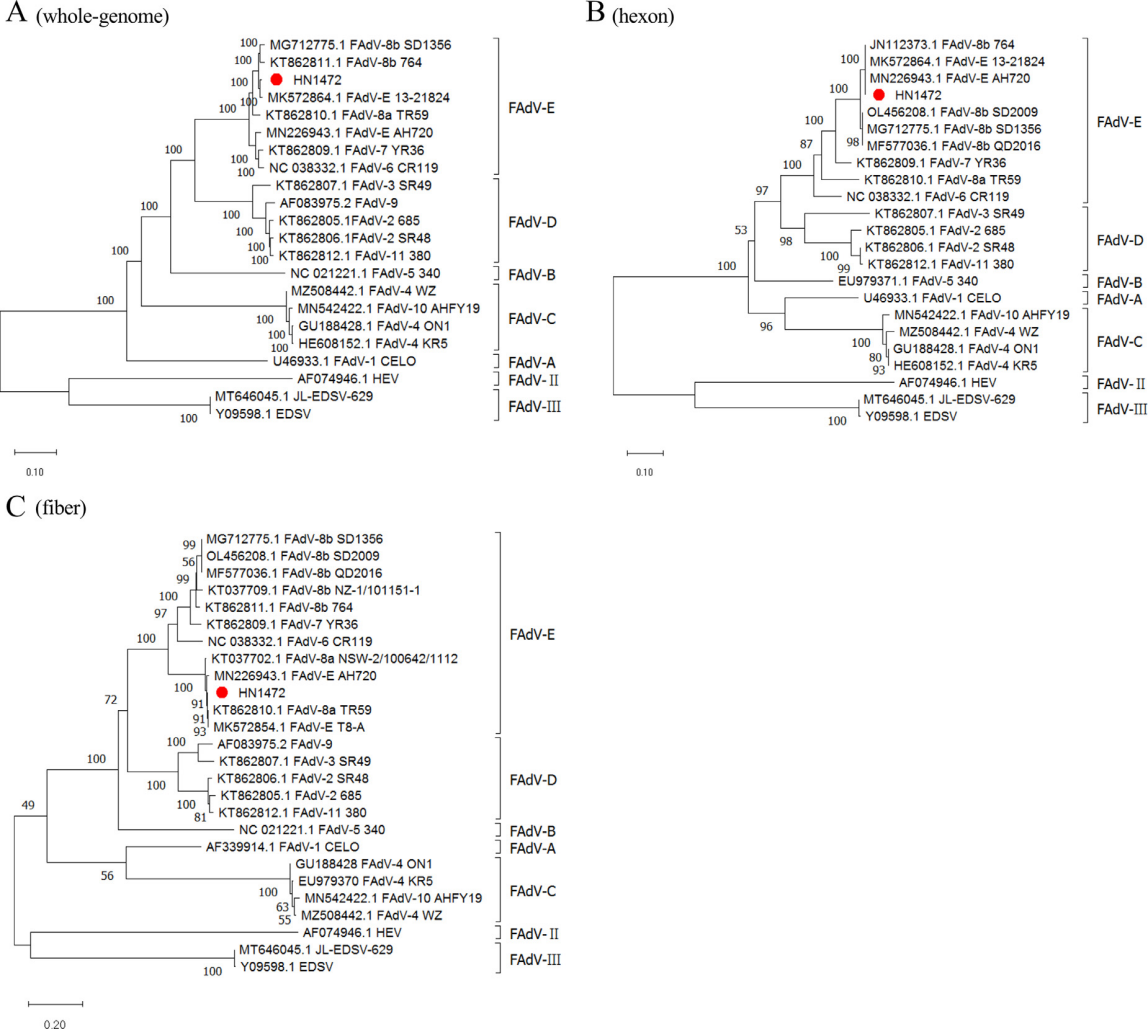
The phylogenetic analysis of the isolate HN1472. The phylogenetic was based on 3 stretches: whole genome sequences (A) and derived amino acid sequences of the entire hexon (B) and fiber gene (C). Strains sequenced in this study are indicated in red; all other sequences were retrieved from GenBank.

The full genome of HN1472 strain was 44,253 nt in length, with a G+C content of 58%. A schematic of the position and relative size of all 34 open reading frames (ORFs) that potentially encode functional proteins is shown in Figure 2. The complete nucleotide sequences were deposited to the GenBank with the accession number (accession number OR975470.1). The sequence was compared with all reference strains represented by FAdV serotypes to reconstruct the phylogeny of the virus. According to phylogenetic analysis based on the whole genome, HN1472 strain was clustered within FAdV-E (Figure 2A), and shared the highest homology (97.3–97.4%) with the France strain FAdV-E 13-21824 (MK572864.1) and China strain FAdV-8b SD1356 (MG712775.1). Furthermore, phylogenetic trees were constructed and analyzed based on the amino acid sequences of hexon and fiber protein. The phylogenetic analysis of the hexon protein sequences indicated that the presently reported FAdV belongs to the same clade and shares a close relationship with FAdV-E sequences from China (8b) (MN226943.1), as depicted in Figure 2B. The fiber protein was additionally characterized by its distinctive sequence pattern, and the sequence of fiber gene had been confirmed with primers of 8a-F/R as described in Table 1. Homology analysis based on proteoprofiles demonstrated that it exhibited the closest relationship with FAdV-8a strains, ranging from 98.7 to 99.6%. In contrast, its association with FAdV-8b isolates ranged only between 78.4% and 79%, as illustrated in Figure 2C. These analyses provided evidence indicating that recombination events occurred between strains of both FAdV-8a and FAdV-8b, resulting in this newly isolated strain.

**Figure 3:**
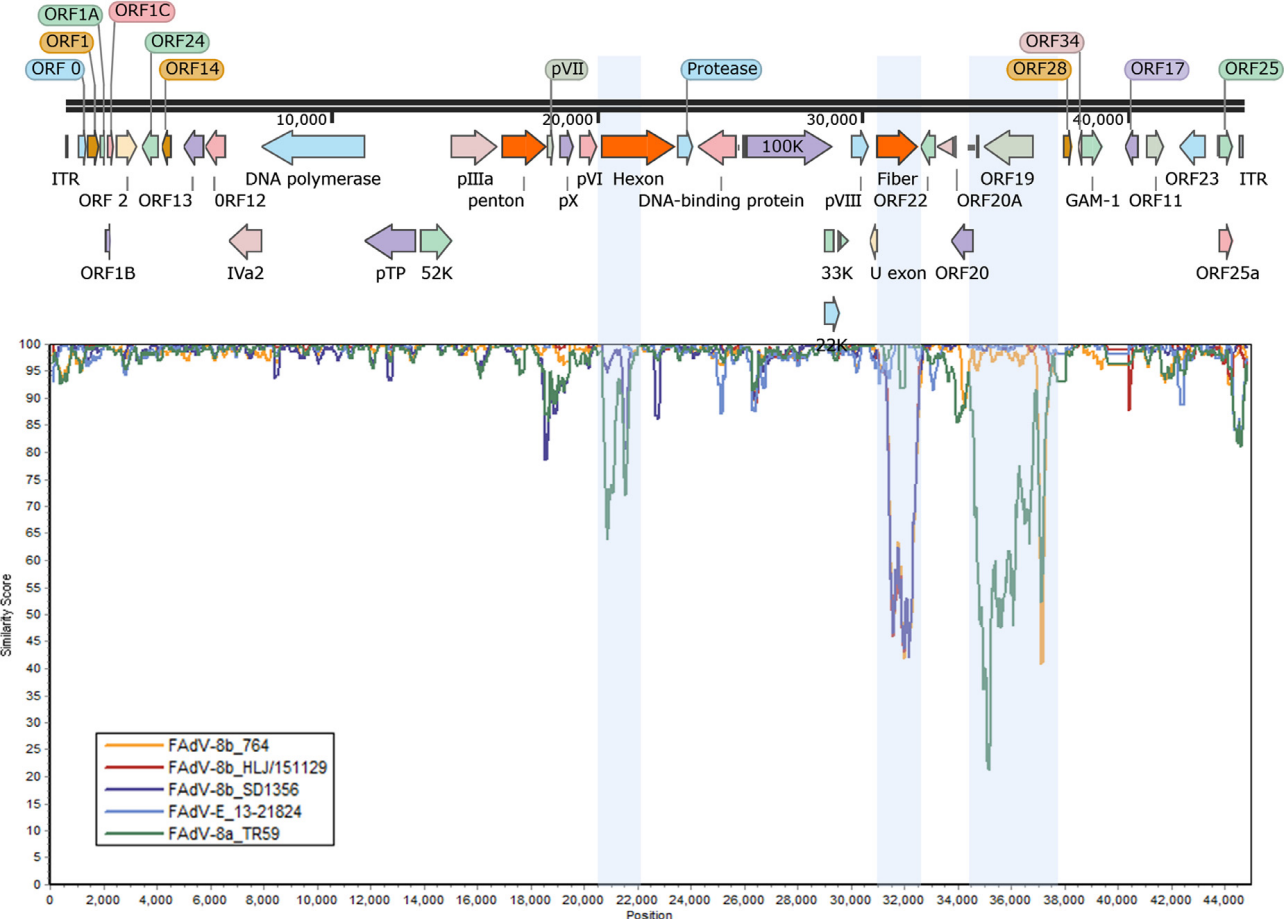
Schematic representation of genome size and organization of the HN1472 isolate (top). The genomic regions encompassed by each cluster are plotted on a comprehensive diversity map. SimPlot analysis was conducted using the HN1472 isolate as the query sequence, while FAdV-8b 764, FAdV-8b HLJ-151129, FAdV-8b SD1356, FAdV-E 13-21824 and FAdV-8a TR59 were considered as potential parental strains. The light blue color indicates the hexon, fiber and ORF19 CDS region.

To further detect possible recombination events within the new isolate HN1472, the genomic sequence analysis of HN1472, FAdV-8b 764, FAdV-8 HLJ/151129, FAdV-8b SD1356, FAdV-E 13-21824 and FAdV-8a TR59 was carried out using the Simplot software (Figure 3). The results showed a peculiar pattern of recombination events in the FAdV-E strains. Compared with these reference strains, 3 recombination signals and breakpoints were observed in the new isolate at 20 297∼21 504 nt, 30 680∼32 142 nt, and 33 722∼36 879 nt. The recombination region between these breakpoints consists of a hexon gene (1 208 nt), a fiber gene (1 463 nt) and an ORF19 gene (3 158nt).

### Pathogenicity Analysis of the Isolate

#### High mortality in HN1472-Inoculated Chickens

**Figure 4:**
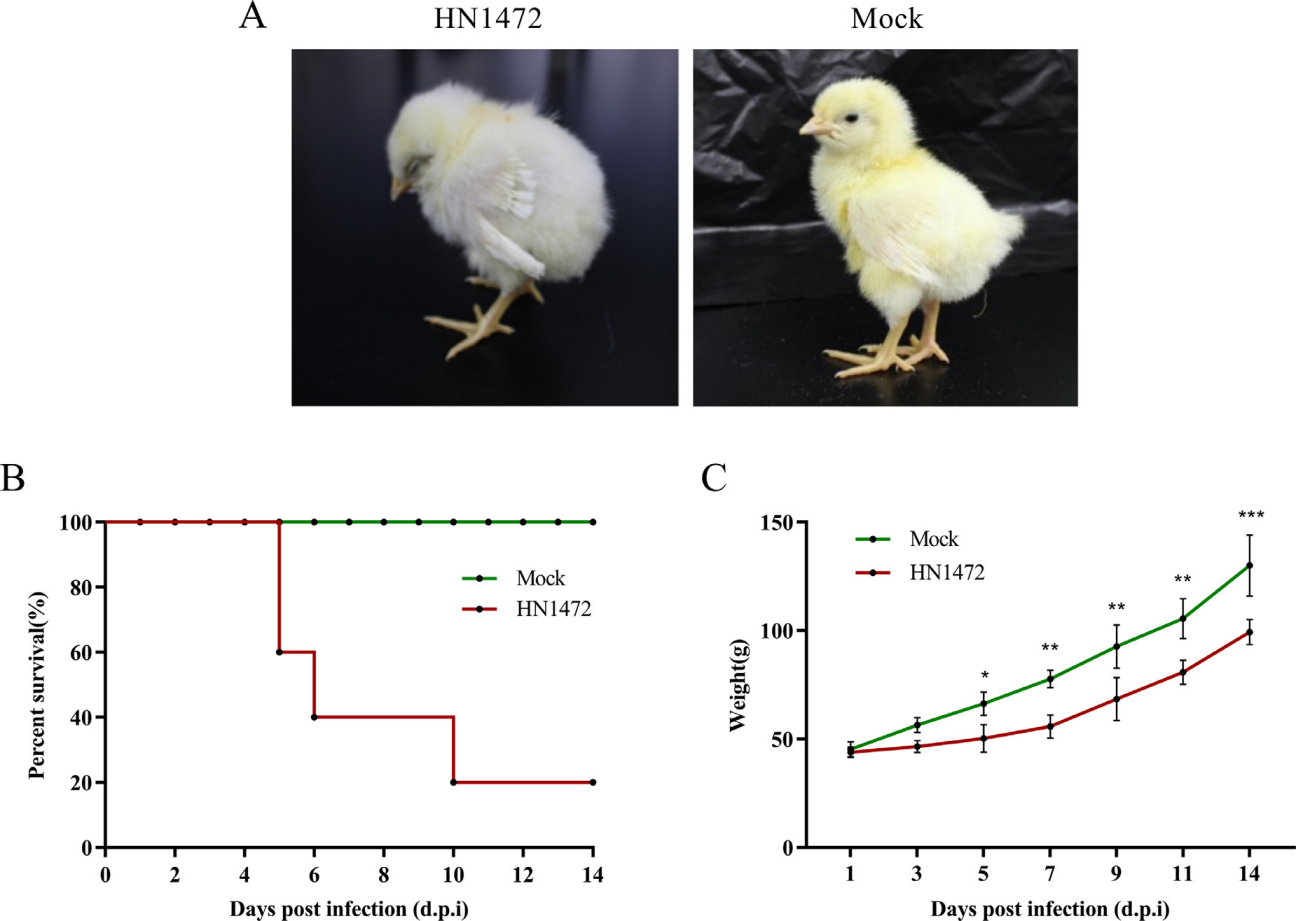
Pathogenicity analysis of the HN1472 isolate. (A) Experimental assessment of the HN1472 virus in SPF chickens. (B) Determination of the Survival rate of SPF chickens infected with HN1472. (C) Evaluation of the percentage differences in body weight among infected birds at 1, 3, 5, 7, 9, 11 and 14 dpc.

The pathogenicity of HN1472 was assessed by infecting 1-day-old SPF chickens, and their clinical signs were monitored for a duration of 14 d. It was observed that the infected chickens exhibited similar clinical signs, including lethargy, retraction of the head, instability of standing, and ruffled feathers after 3 d postinfection (**dpc**) (Figure 4A). The experimental group displayed symptoms of illness with a total of 4 deaths occurring at 5 dpc. The peak mortality rate was observed between 4 dpc and 10 dpc. From 10 dpc onwards, no overt clinical signs were detected (Figure 4B). However, at the end of the observation period, all chickens in the experimental group showed reduced growth when compared to the control chickens (Figure 4C).

### Severe Gross Pathology and Microscopic Lesions Induced by HN1472

**Figure 5:**
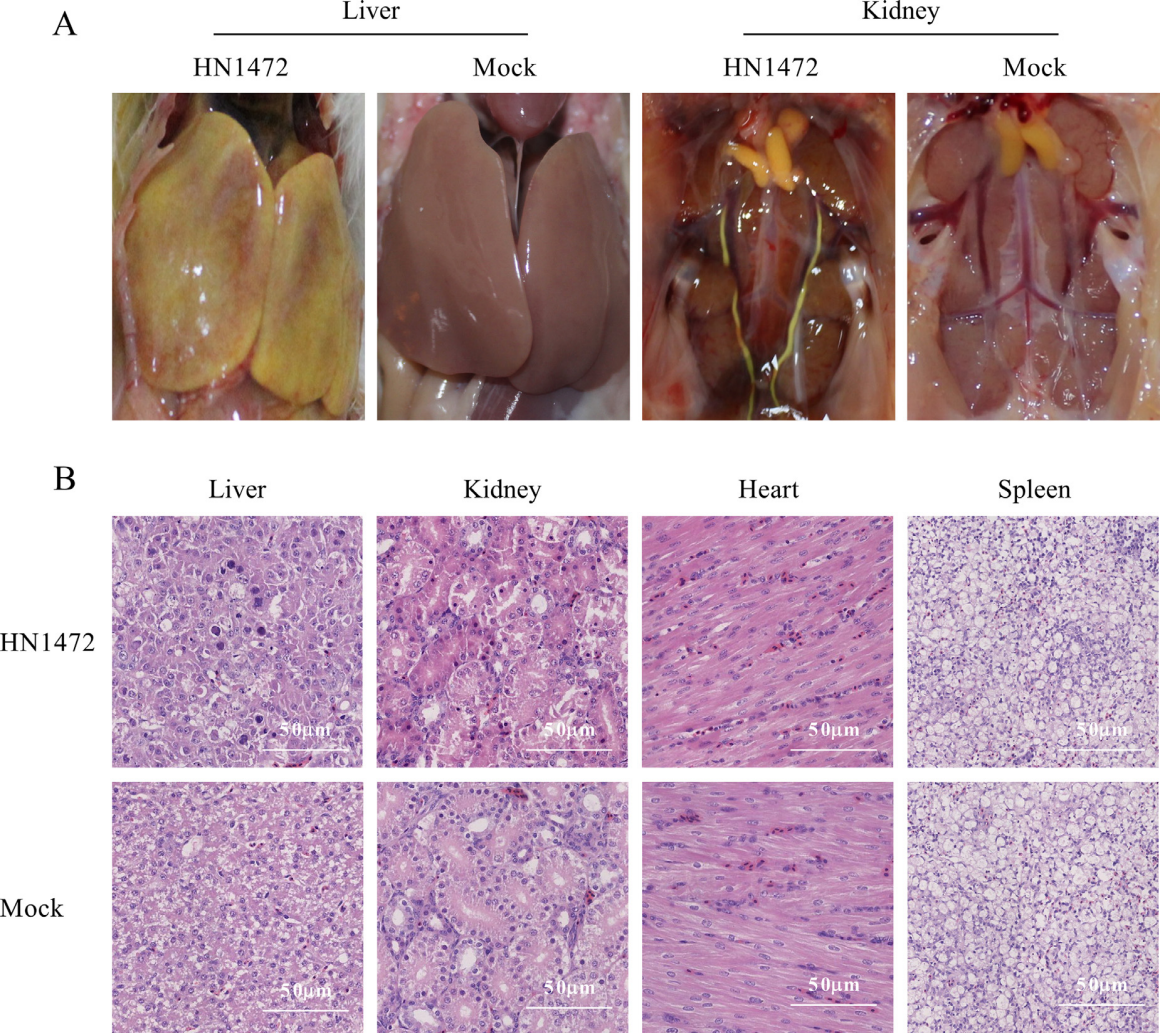
Gross lesions and histopathology changes in chicken tissues. (A) Gross lesions in the livers and kidney infected with HN1472. (B) Histopathology of intramuscularly inoculated chickens in liver, kidney, heart and spleen tissues (**H&E**).

Histopathological analysis revealed extensive pathological damages in various tissues of the infected group chickens in (Figure 5A). In the HN1472 challenge group, a distinct yellow-green appearance was observed, indicting severe liver necrosis Additionally, in comparison to the PBS group, kidney enlargement accompanied by yellow urate deposits in the ureter was observed. The liver tissue exhibited typical inclusion body hepatitis lesions, characterized by lymphocyte infiltration, vacuolar degeneration, and granular degeneration. Alkaline inclusion bodies were observed in the liver nucleus, consistent with typical symptoms of IBH infection. In the kidney, marked hemorrhage congestion in the renal interstitium, necrosis of renal tubular epithelial cells, and lymphocyte infiltration were presented. Cavitation degeneration and gap widening were observed in the heart tissues. Conversely, no significant histopathological damage was found in the tissues of chickens in the control group (Figure 5B). According to histopathological analysis, the average lesion scores of the liver, kidney, heart, and spleen are 2.4, 1.8, 0.6, and 0.2, respectively.

### The Analysis of Viral Load, Shedding and Distribution in Chickens

**Figure 6:**
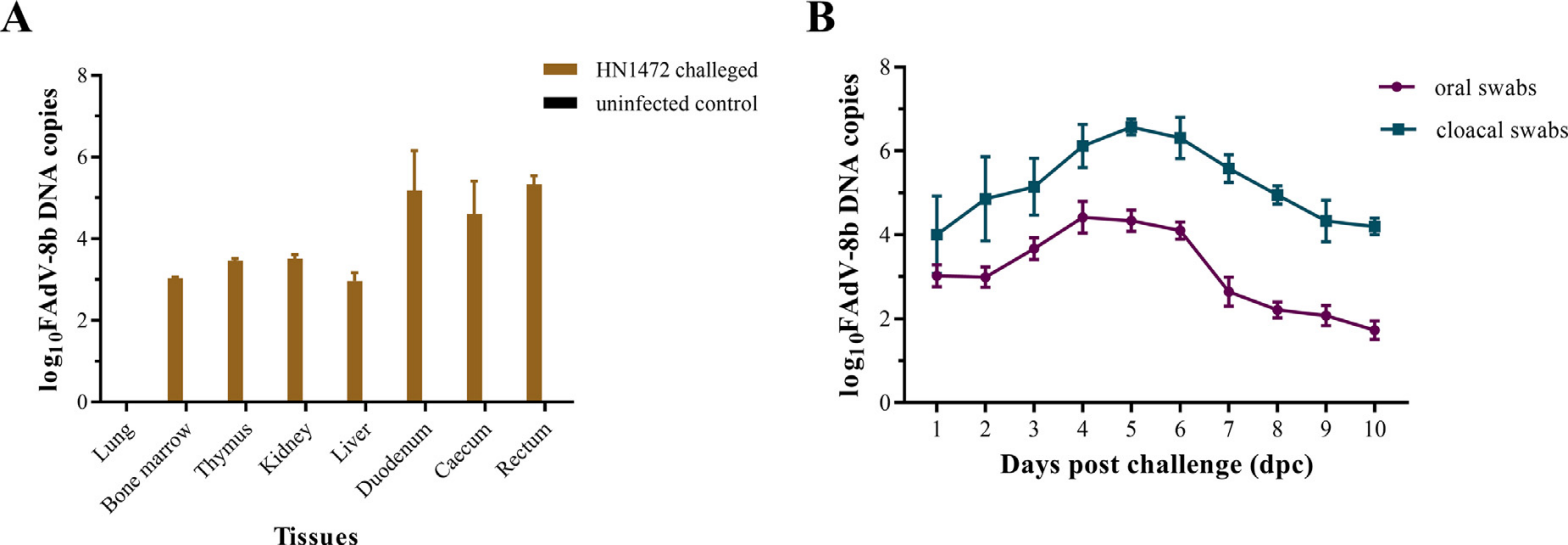
Detection of virus in SPF chickens. (A) Quantification of viral loads in various chicken tissues following inoculation with HN1472 at 3 dpc. (B) Assessment of viral shedding through oral and cloacal swabs among different groups of chickens.

The copy number of FAdV-8b HN1472 in each internal organ of infected chickens was assessed using real-time PCR. In order to investigate the distribution of the FAdV-8b virus across different tissues, 3 chickens from the experimental group were subjected to necropy at 3 dpc. The levels of viral DNA in the liver, gizzard, cecal tonsil, bursa, spleen, kidney, and thymus were quantified (Figure 6A). No viral DNA was detected in the tissues of control chickens throughout the duration of the experiment. At 3 dpc, a high viral load was observed in the heart, liver, spleen, kidney, thymus, and bursa. Notably, the viral load in the liver was significantly higher than that in other tissues; closely followed by that in gizzard tissue. The amount of virus shed through cloacal and throat swabs from chickens inoculated with HN1472 reached its peak at 5 dpc before gradually declining (Figure 6B).

## DISCUSSION

Inclusion body hepatitis is widely recognized as a disease of significant economic importance worldwide. The role of FAdV-8b as a causative agent of IBH has also been extensively documented in numerous countries. In China, the serotypes isolated from IBH cases encompass type 8a, -8b, and -11; however, most of these isolates have been obtained from chickens exhibiting either mild or no clinical signs. This study focused on the isolation of recombinant FAdV-E HN1472 strains from IBH outbreaks and their clinical relevance. Specifically, we investigate the genomic organization and relationship with other FAdVs, as well as their pathogenicity in SPF chickens. By obtaining the complete genome sequence of FAdV-8b, our aim is to contribute to a comprehensive understanding of its genetic content, phylogeny, and evolution within the Aviadenovirus genus.

The strain HN1472 was subjected to whole-genome characterization and classified as a member of the E species within FAdV-8b, exhibiting high homology based on full genome and hexon gene analysis. Recombination analysis and phylogenetic analysis revealed that this new isolate is a recombinant strain resulting from recombination events between FAdV-8a and FAdV-8b. Interestingly, the major recombination regions and genes identified in HN1472 were consistent with the recombinant chimeric pattern previously reported by Schachner et al. (2019), including the hexon gene, fiber gene, and ORF19. Notably, genetic variability in the fiber gene was found to be greater than that observed in the hexon gene, which is also a phenomenon observed in human adenoviruses (Schachner et al., 2016). Recombination events occurring between genomes of aviadenoviral species are known to have significant impacts on their biological properties.

The HN1742 strain, characterized by typical IBH symptoms, exhibited a remarkably high virulence and caused significant mortality in SPF chicks when administered via the intramuscular route. HN1742 demonstrated the ability to replicate and proliferate in multiple organs, with the liver and intestine of chickens harboring the highest number of genomic copies, consistent with previous findings (Vera-Hernández et al., 2015). Pathogenicity analysis revealed that experimentally infected chickens experienced moderate to severe clinical signs and an 80% mortality rate due to HN1742 infection. It is worth noting that natural recombination between FAdV-E strains has been confirmed in several studies; however, these recombinants were considered moderately pathogenic without causing clinical signs or mortality (Grgić et al., 2011; Huang et al., 2019). Furthermore, there have been reports indicating that mutations within adenoviruses can lead to increased virulence of FAdV strains (Pan et al., 2017; Chen et al., 2019; Lv et al., 2020). Therefore, further investigations are required to elucidate the mechanisms underlying the pathogenicity, molecular variations, and virulence determinants of these novel FAdV recombinants.

Differences in the knob domain of the fiber gene, as well as in the L1 loop domain of the hexon gene, have been implicated in variations in tissue tropism and virulence (Yuhan et al., 2018). However, due to the absence of virulence markers within the sequence, direct sequence comparison of FAdV-8b fiber gene alone cannot distinguish between different FAdV prototypes (Sabarudin et al., 2021). Therefore, further investigation is required to elucidate the molecular mechanism differences between highly virulent strains and nonvirulent strains of FAdV-8. Although HN1472 was identified as a causative agent for IBH, its ability to evade existing vaccine immunity may be determined by mutations and recombination events occurring within both hexon and fiber genes. This poses a significant threat to poultry farms and highlights the urgent need for developing new FAdV vaccines.

Following the global literature, species C, which represents type 4, remains unequivocally predominant in China (Cui et al., 2020; Niczyporuk et al., 2021). Inclusion body hepatitis sporadically affects broilers without causing significant epidemics in other chicken varieties. However, given the high mortality rate associated with the HN1472 strain, greater attention should be devoted to IBH-induced adenoviruses. Further research is warranted to investigate the pathogenic mechanism and develop vaccines for FAdV-8b.

## ACKNOWLEDGMENTS

This work was supported by grants from The National Natural Science Fundation of China (32102653), Henan open competition mechanism to select the best candidates Program (211110111000), and the key scientific research projects of colleges and universities in Henan Province (23B230002).

Ethics Approval: The SPF chickens were subjected to humane procedures and maintained in accordance with protocols approved by the HENAU animal ethics committees on Animal Care (HNND2021030231).

Data and Model Availability Statement: None of the data were deposited in an official repository. The data that support the findings of this study are available from the corresponding author upon reasonable request.

Author Contributions: Conceptualization, Investigation: Yapeng Song, Zongmei Huang; Methodology: Wenjie Sun, Xiaonan Song, Yang Wang, Lin Liu; Writing-original draft: Wenjie Sun, Lin Liu; Writing–review and editing: Yapeng Song, Qiang Wei; Validation: Wenming Gao; Supervision, Funding acquisition: Yapeng Song, Xinsheng Li.

## DISCLOSURES

The authors declare that there have no conflicts of interest.
